# CCT7 Regulates TRAF6‐Mediated Autophagy to Promote Posttraumatic Joint Contracture

**DOI:** 10.1111/jcmm.71051

**Published:** 2026-04-07

**Authors:** Yutai Li, Wenhui Zhang, Wenbin Xu, Yuliang Huang, Honghua Cai, Nanwei Zhang, Tangzhao Liang, Guihua Liu

**Affiliations:** ^1^ Department of Orthopaedics Huizhou Central People's Hospital Huizhou China; ^2^ Hui Zhou‐Hong Kong Bone Health Joint Research Center Huizhou China; ^3^ Huizhou Central People's Hospital Academy of Medical Sciences, Huizhou Central People's Hospital Huizhou China; ^4^ The Third Affiliated Hospital, Sun Yat‐Sen University Guangzhou China

**Keywords:** autophagy, CCT7, fibrosis, posttraumatic joint contracture, TRAF6

## Abstract

Posttraumatic joint contracture (PTJC) is a prevalent complication of joint injury, characterised by marked reductions in both active and passive joint motion. Previous studies have indicated that the chaperonin‐containing T‐complex polypeptide (CCT) is involved in fibrotic processes. Thus, this study aims to explore the role of CCT7 in PTJC and clarify its underlying regulatory mechanisms. We found that CCT7 expression was significantly upregulated in PTJC joint tissues and in fibroblasts stimulated with TGF‐β1. Knockdown of CCT7 markedly reduced the expression of α‐SMA and COL‐I and suppressed fibroblast migration, while simultaneously promoting autophagy, as indicated by increased LC3‐II and Beclin‐1 levels and decreased p62 expression, thereby alleviating joint fibrosis. Interestingly, CCT7 interacted with TRAF6 to facilitate its ubiquitination and degradation. Knockdown of TRAF6 reversed the promoting effect of CCT7 knockdown on autophagy in fibroblasts and exacerbated fibrosis. Consistently, in vivo experiments demonstrated that CCT7 knockdown improved joint range of motion, reduced the expression of fibrosis‐related proteins, enhanced autophagy and ultimately alleviated fibrosis.

## 1 Introduction

Posttraumatic joint contracture (PTJC) is a common complication following joint injury, characterised by markedly reduced active and passive range of motion [1, 2]. The central pathological hallmark of PTJC is joint fibrosis, a progressive process driven by intra‐articular adhesions and inflammatory responses that promote extracellular matrix (ECM) deposition and fibrotic tissue formation within the joint cavity [3, 4]. After trauma, transforming growth factor‐β1 (TGF‐β1) and pro‐inflammatory cytokines promote fibroblast activation, collagen cross‐linking and tissue contraction [5, 6, 7]. These changes result in the loss of joint compliance and the development of fibrotic adhesions, leading to joint stiffness and dysfunction [8, 9].

CCT7, also known as TCP1 subunit 7 with co‐chaperone properties, is an essential component of the molecular chaperone TCP‐1 complex (CCT) involved in protein folding and cellular stress regulation [10]. Recent studies have revealed that the CCT complex may contribute to tissue fibrosis by modulating collagen synthesis and fibroblast function [11, 12]. In a study conducted in a rat model of PTJC, the biological function of the CCT subunit in fibroblasts was identified, and a significant reduction of CCT6b was found in activated fibroblasts. Notably, overexpression of CCT6b significantly inhibited fibroblast activity, leading to reduced expression of α‐SMA and COL‐I, accompanied by a decrease in the fibrosis‐associated chaperone protein CCT7 [13]. Similarly, another study compared the wound healing processes in foetal and adult rabbit skin, observing scarless healing in the former and scar formation in the latter. They further discovered that both the average protein and mRNA levels of CCT7 in adult rabbit fibroblasts were significantly higher than those in foetal fibroblasts, indicating a positive correlation between CCT7 expression and the occurrence of fibrosis [14]. These findings suggest that an elevated level of CCT7 may be associated with an exacerbation of the fibrotic process, and that modulation of CCT subunit expression could serve as a potential strategy for intervening in fibrosis and PTJC.

Recent evidence suggests that autophagy is intricately linked to the regulation of fibrosis. In fibrotic diseases, impaired or dysregulated autophagy can promote fibroblast activation, myofibroblast differentiation and excessive ECM deposition. For example, insufficient autophagic activity in hepatic and pulmonary fibrosis is associated with enhanced collagen production and tissue scarring. Restoration of autophagic flux has been shown to mitigate these fibrotic responses by limiting fibroblast contractility and ECM accumulation. Tumour necrosis factor receptor‐associated factor 6 (TRAF6), a key member of the TRAF protein family, plays crucial roles in immune regulation, inflammatory responses, tumorigenesis and skeletal metabolism [15, 16, 17]. Recent studies have revealed that TRAF6 is not only a key regulator of inflammatory signalling but also participates in fibrosis regulation by mediating autophagy through its E3 ubiquitin ligase activity. TRAF6 can promote autophagy by catalysing K63‐linked ubiquitination of autophagy‐related proteins such as Beclin‐1, thereby facilitating autophagosome formation. Dysregulation of TRAF6 activity can disrupt autophagic signalling and contribute to pathological tissue remodelling. However, the role of TRAF6‐mediated autophagy in PTJC remains unclear. This study aims to clarify the roles of CCT7 and TRAF6 in PTJC and uncover the underlying molecular mechanisms.

## 2 Materials and Methods

### 2.1 Rat PTJC Model

Male Sprague–Dawley (SD) rats (180–220 g) were purchased from Guangzhou Ruige Biological Technology Co. Ltd. and housed in a specific pathogen‐free (SPF) environment with a 12 h light–dark cycle, with free access to food and water. All experimental procedures were approved by the Ethics Committee of Guangzhou Seyotin Biotechnology Co. Ltd (SYT2024112).

The PTJC model was established based on previous studies [18, 19] with the following procedures: The rats were anaesthetised with 50 mg/kg sodium pentobarbital and secured on a surgical table. A midline skin incision was made over the knee joint, followed by a lateral parapatellar arthrotomy. After flexing the knee joint, a drill was used to create a hole through the femur, into which a Kirschner wire was inserted for positioning. Subsequently, a longitudinal incision was made at the tibial tuberosity, and a hole was drilled through the tibia along the Kirschner. The Kirschner was then passed through the tibia to fix the knee joint at 45°. After repositioning the patellofemoral joint, the muscles and skin were closed layer‐by‐layer with sutures. In the treatment groups, si‐NC or si‐CCT7 was injected into the joint cavity after sutured. Postoperatively, rats were returned to their cages and allowed unrestricted movement. After 4 weeks, the rats were euthanised, and the internal fixation device was removed. The range of motion (ROM) of the knee joint was measured, and joint tissues were collected for further analysis.

### 2.2 Cell Culture

Based on prior studies [19, 20], following euthanisation, SD rats were placed under strictly aseptic conditions, and the posterior joint capsule tissues of their knee joints were carefully dissected. The isolated tissues were rinsed thoroughly three times with phosphate‐buffered saline (PBS), with all visible adipose tissue meticulously excised using sterile forceps. The cleaned posterior joint capsule tissues were cut into fine fragments of approximately 1–2 mm^3^ and evenly distributed across sterile cell culture dishes. An appropriate volume of Dulbecco's Modified Eagle Medium (DMEM) supplemented with 10% foetal bovine serum (FBS) was added to each dish, and the cultures were maintained in a humidified incubator at 37°C with 5% CO2. Subculture was performed once the confluency of fibroblasts migrating from the edges of tissue fragments reached 80%–90%. During the culture process, TGF‐β1 was added to the medium at a concentration of 10 ng/mL. The cells were treated for 24 h before being utilised for subsequent experiments.

### 2.3 Transwell Assay

Cells were prepared as a single‐cell suspension at a concentration of 1 × 10^5^/mL and seeded into the upper chamber of a Transwell insert. The lower chamber was filled with 500 μL of DMEM medium containing 10% foetal bovine serum. After incubation for 24 h at 37°C in a 5% CO2 atmosphere, cells were fixed with 4% formaldehyde and stained with 0.1% crystal violet. The number of migrated cells was then observed and quantified to assess cell migratory capacity.

### 2.4 Transmission Electron Microscope (TEM)

Fibroblast samples were collected and fixed with 2.5% glutaraldehyde at 4°C overnight. Following fixation, the samples were subjected to dehydration, embedding and ultrathin sectioning to a thickness of 70 nm. The sections were double stained with uranyl acetate and lead citrate and then observed under a TEM to visualise and photograph the autophagosome structures within the cells.

### 2.5 Masson Staining

After deparaffinising the joint tissue sections in water, the staining procedure was carried out sequentially. Initially, the sections were stained with acid fuchsin solution for 5–10 min to render the nuclei and cytoplasm red. Subsequently, they were differentiated in a 1% tungstophosphoric acid solution for 1–2 min to lighten the colour. Next, the sections were stained with brilliant blue solution for 5–10 min to stain collagen fibres blue. Finally, the sections were differentiated in a 1% glacial acetic acid solution for 1–2 min to terminate the reaction. Following staining, the sections were dehydrated through a graded ethanol series (70%–100%), cleared in xylene and mounted with neutral gum. The stained sections were then examined and photographed under an optical microscope.

### 2.6 Immunohistochemistry

Paraffin sections were deparaffinised and rehydrated, then subjected to antigen retrieval in citrate buffer (pH 6.0) using microwave treatment. Sections were treated with 3% hydrogen peroxide for 10 min at room temperature, followed by blocking with 5% normal goat serum for 30 min. Primary antibodies against α‐SMA and COL‐I were applied and incubated overnight at 4°C. After washing with PBS, secondary antibodies were added and incubated for 1 h at room temperature. Sections were then stained with DAB for 5 min and counterstained with haematoxylin for 1 min. Finally, the sections were observed and photographed under a light microscope.

### 2.7 Immunofluorescence

Cells or tissue sections were fixed in a 4% paraformaldehyde solution. After washing with PBS, the samples were blocked with 5% normal goat serum in PBS for 1 h at room temperature. Primary antibodies targeting CCT7 (Proteintech, 1:200, 15,994–1‐AP), LC3 (Proteintech, 1:500, 14,600–1‐AP), p62 (Proteintech, 1:200, 31,403–1‐AP), Beclin‐1 (Proteintech, 1:400, 11,306–1‐AP), α‐SMA (Abcam, 1:400, ab7817), COL‐I (Proteintech, 1:500, 66,761–1‐Ig) and TRAF6 (Abcam, 1:400, ab40675) were applied and incubated overnight at 4°C. On the following day, the samples were washed three times with PBS, followed by incubation with fluorophore‐conjugated secondary antibodies for 1 h at room temperature in the dark. Finally, nuclei were counterstained with DAPI, and the samples were mounted and imaged under a fluorescence microscope.

### 2.8 Real‐Time Quantitative PCR (qPCR)

Total RNA was extracted from cell and tissue samples using TRIzol reagent. The concentration and purity of the RNA were subsequently measured. 1 μg of RNA was reverse transcribed into cDNA using a reverse transcription reaction. The synthesised cDNA was used as a template for qPCR analysis, performed with a SYBR Green qPCR kit. The relative expression levels of target genes were calculated using the 2^−ΔΔCt^ method. The primer sequences are listed in (Table 1).

**TABLE 1:** Primers sequence.

Genes	Forward (5′‐3′)	Reverse (5′‐3′)
CCT1	TGCAAGGAAGCAGTGCGATA	GCAAGCACAGCGTCTACCAC
CCT2	CCTGGCAGACTCCTACCTTG	GCAACCTTGGCTGTGGAATC
CCT3	CATCACAGAAAAGGGCATCTCG	TCTCTCAGTTCCTCGGGTCG
CCT4	TGCAACTGAGCGACAGAGAA	CGTGGCTGGATCGATCACTTTC
CCT5	TCTCGTCTCATGGGGCTTGA	GCACCATCGTTGGTCACAG
CCT6	GCCCATTGACCTCTTCATGGTT	GTTGCATGTGAGGATGTAGGCA
CCT7	AACCCCAAGATTGCCCTCTTAA	GAGAATGTTCCACTCGGCATCA
TGF‐β1	GCTGAACCAAGGAGACGGAA	TCATGTCATGGATGGTGCCC
α‐SMA	CCAGAGGAGCATCCGACCTT	GCACAGCCTGAATAGCCACA
COL‐I	ATGGCCAACCTGGTGCGAAA	ACCAGCAGCACCAGGGAAAC
TRAF6	GTGCCCATGCCGTATGAAGA	GGAGCTGTTGGGCAGTCTAG
GAPDH	GCTGAGAATGGGAAGCTGGT	GACGCCAGTGGACTCCACGA

### 2.9 Coimmunoprecipitation (Co‐IP)

Total protein was extracted from cell samples and lysed with RIPA buffer. Protein samples were incubated with 2 μg of anti‐CCT7 or anti‐TRAF6 antibody overnight at 4°C. Subsequently, 50 μL of protein A/G agarose beads was added, and the mixture was further incubated for 2 h at 4°C to allow antibody binding to the beads. The beads were then washed three times with PBS, followed by centrifugation at 10,000 rpm for 1 min each time to remove unbound proteins. Finally, the immunoprecipitated proteins were resuspended in 5× loading buffer, boiled at 100°C for 5 min to denature the proteins and prepared for subsequent Western blot analysis.

### 2.10 Western Blot

Cell and tissue samples were harvested and lysed with RIPA buffer to obtain total protein extracts. Protein concentrations were measured using a BCA protein assay kit. Equal amounts of protein samples were subjected to SDS‐PAGE electrophoresis for separation, followed by transfer onto PVDF membranes. The membranes were blocked with 5% skim milk for 2 h at room temperature. Subsequently, primary antibodies against TGF‐β1 (Abcam, 1:1000, ab215715), α‐SMA (Abcam, 1:10000, ab7817), COL‐I (Proteintech, 1:5000, 66,761–1‐Ig), p62 (Proteintech, 1:5000, 31,403–1‐AP), Beclin‐1 (Proteintech, 1:10000, 11,306–1‐AP) and TRAF6 (Abcam, 1:5000, ab40675) were applied and incubated overnight at 4°C. After washing, the membranes were incubated with secondary antibodies for 1 h at room temperature. Chemiluminescent detection was performed, and images were captured using a gel imaging system. The band intensities were quantified using ImageJ software.

### 2.11 Statistical Analysis

Data were analysed using GraphPad Prism version 9.0 (GraphPad Software, San Diego, CA, USA). All experiments were performed at least three times independently, and results are presented as the mean ± standard deviation (SD). To compare two groups, an unpaired two‐sided Student's *t*‐test was utilised. For comparisons involving multiple groups, one‐way ANOVA was employed. A *p* < 0.05 was considered statistically significant.

## 3 Results

### 3.1 CCT7 Is Significantly Upregulated in the Joint Capsule of Rats Following PTJC

**FIGURE 1:**
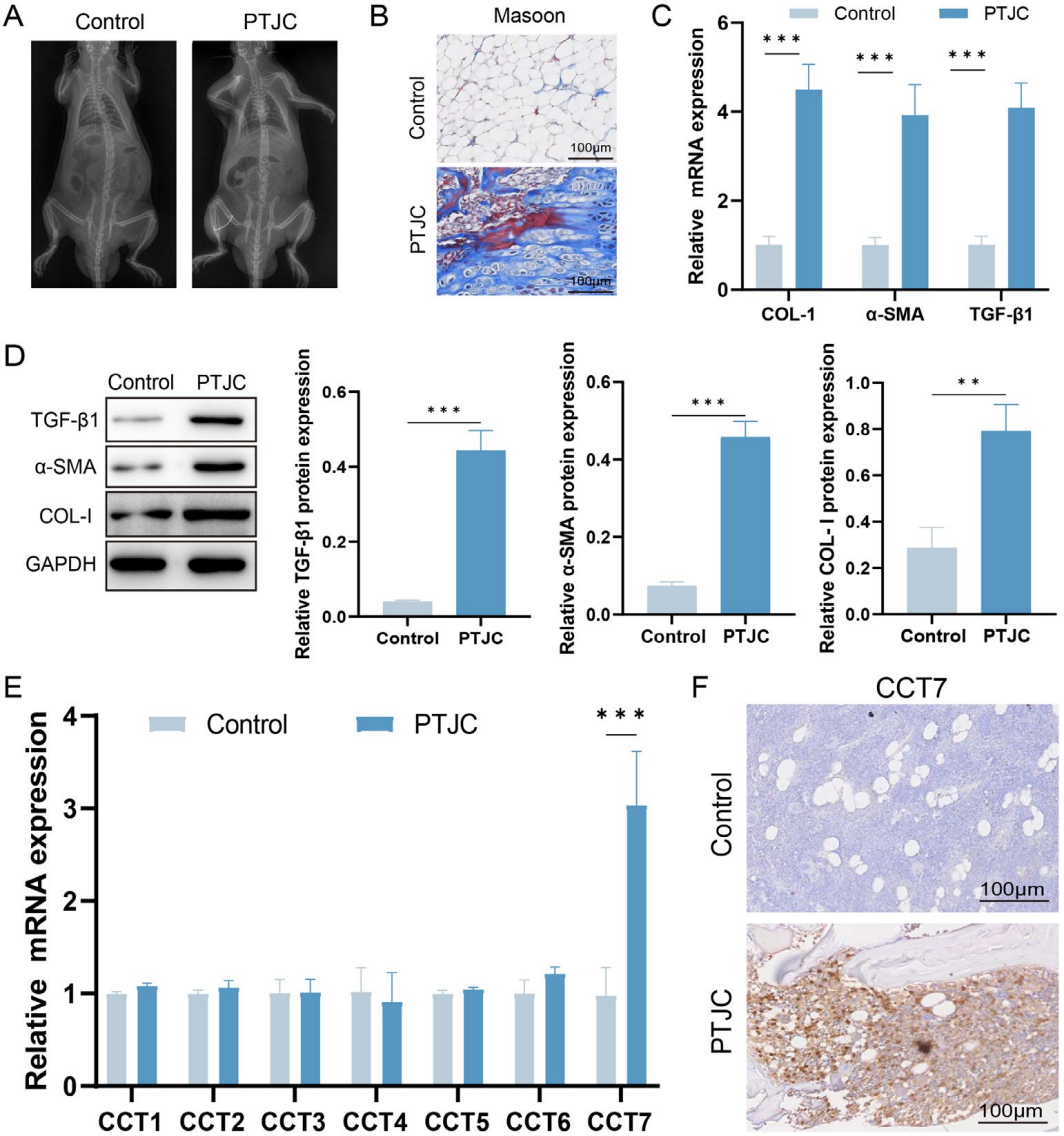
CCT7 expression is significantly upregulated in a rat model of PTJC. (A) Representative images of X‐ray images. (B) Masson's trichrome staining of joint capsule tissues from control and PTJC model groups. (C) qPCR analysis of COL‐I, α‐SMA and TGF‐β1 mRNA expression in joint tissues. (D) Western blot analysis of TGF‐β1, α‐SMA and COL‐I protein expression in joint tissues. (E) qPCR analysis of CCT1, CCT2, CCT3, CCT4, CCT5, CCT6, CCT7 mRNA expression in joint tissues. (F) Immunohistochemical staining of CCT7 in joint tissues. **p < 0.01, ***p < 0.001.

To study the role of CCT7 in PTJC, the joint capsule was sutured at a 45° angle to establish a PTJC rat model, and (Figure 1A) presents the X‐ray image. Masson's trichrome staining revealed significant collagen deposition in the joint capsule of the model group compared to the control group (Figure 1B). Western blot and qPCR analyses revealed increased expression of TGF‐β1, α‐SMA and COL‐I in the model group compared to the control group (Figure 1C,D). The qPCR results indicated that among the CCT1, CCT2, CCT3, CCT4, CCT5, CCT6 and CCT7 genes, only CCT7 was specifically upregulated in the model group, while no significant changes were observed in the other genes (Figure 1E). Immunohistochemical results also confirmed that CCT7 expression was increased in the model group compared with the control group (Figure 1F). These findings indicate that the joint capsule exhibits significant fibrosis following PTJC in rats, which may be associated with the marked upregulation of CCT7.

### 3.2 Knockdown of CCT7 Reduces the Expression of Fibrotic Markers in TGF‐β1‐Stimulated Fibroblasts

**FIGURE 2:**
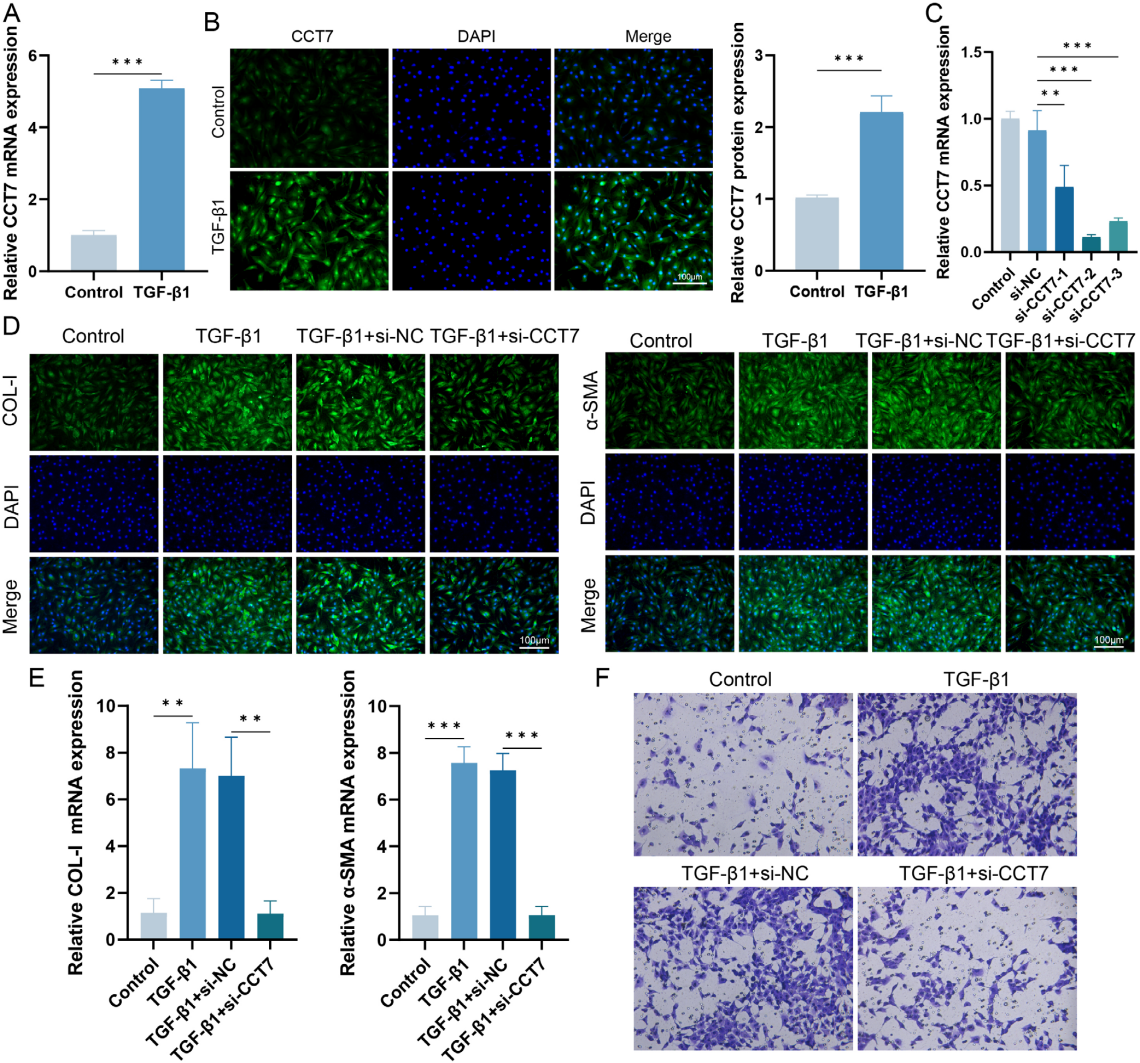
CCT7 knockdown reduces the expression of fibrotic markers in fibroblasts stimulated with TGF‐β1. (A) qPCR analysis of CCT7 expression in fibroblasts treated with TGF‐β1. (B) Immunofluorescence staining of CCT7 in fibroblasts treated with TGF‐β1. (C) qPCR analysis showing the expression levels of CCT7 in fibroblasts transfected with *CCT7* siRNA. (D) Immunofluorescence staining of COL‐I and α‐SMA in fibroblasts under different treatment conditions. (E) qPCR analysis of COL‐I and α‐SMA mRNA expression in fibroblasts under different treatment conditions. (F) Transwell migration assay showing the migratory capacity of fibroblasts under different treatment conditions. **p < 0.01, ***p < 0.001.

To further investigate the role of CCT7 in fibrosis, we performed in vitro experiments using fibroblasts treated with TGF‐β1. The expression level of CCT7 was detected by qPCR and immunofluorescence. Results showed that TGF‐β1 treatment led to the upregulation of CCT7 expression compared with the control group (Figure 2A,B). Subsequently, we evaluated the efficiency of CCT7 gene knockdown by qPCR (Figure 2C). Following successful CCT7 knockdown, we investigated its effect on the expression of fibrotic markers in fibroblasts. Immunofluorescence staining and qPCR analysis revealed that CCT7 knockdown significantly inhibited the expression of fibrotic markers α‐SMA and COL‐I in TGF‐β1‐stimulated fibroblasts (Figure 2D,E). Additionally, Transwell assays demonstrated that the promotive effect of TGF‐β1 on fibroblast migration was attenuated after CCT7 knockdown (Figure 2F). These findings indicate that CCT7 knockdown can inhibit the profibrotic effect of TGF‐β1 on fibroblasts.

### 3.3 Knockdown of CCT7 Enhances Autophagy in Fibroblasts Stimulated With TGF‐β1

**FIGURE 3:**
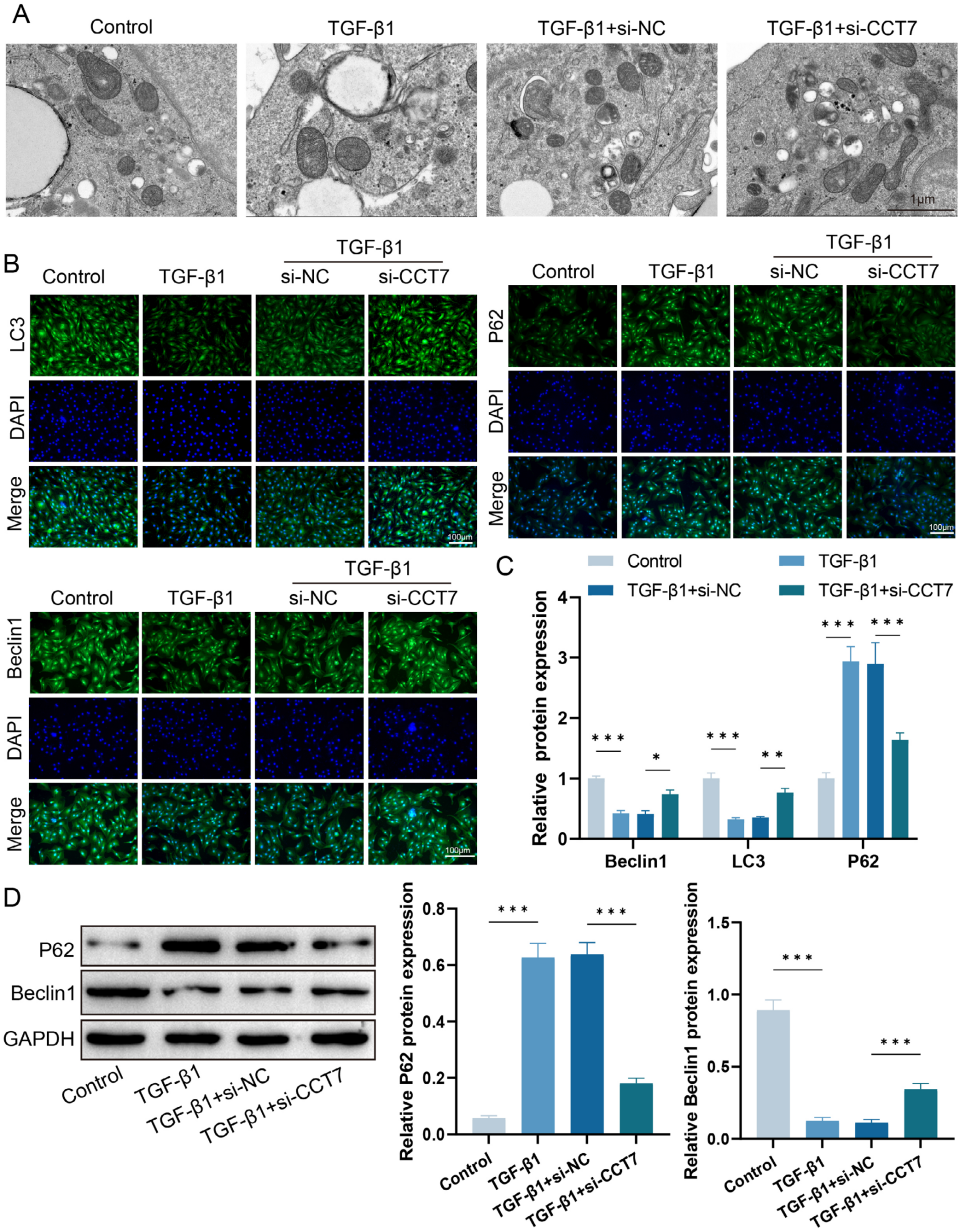
CCT7 knockdown enhances autophagy in TGF‐β1‐stimulated fibroblasts. (A) Transmission electron microscopy images of autophagosomes in fibroblasts under different treatment conditions. (B‐C) Immunofluorescence staining of LC3, p62 and Beclin‐1 in fibroblasts under different treatment conditions. (D) Western blot analysis of p62 and Beclin‐1 expression levels under different treatment conditions. *p < 0.05, **p < 0.01, ***p < 0.001.

To clarify whether the pro‐fibrotic effect of CCT7 is associated with autophagy, we detected the autophagic activity of fibroblasts stimulated with TGF‐β1. TEM results showed that the number of autophagosomes was decreased in TGF‐β1‐treated fibroblasts, while CCT7 knockdown reversed this effect (Figure 3A). Detection of autophagy‐related proteins revealed that TGF‐β1 induction reduced the expression of LC3‐II and Beclin‐1 in fibroblasts, accompanied by a significant increase in p62 expression, indicating autophagic impairment. However, CCT7 knockdown remarkably reversed this phenomenon: the increased expressions of LC3‐II and Beclin‐1, as well as the decreased p62 level, suggested that knockdown of CCT7 enhanced autophagy in fibroblasts (Figure 3B–D).

### 3.4 CCT7 Regulates the Stability of TRAF6 Through Ubiquitination

**FIGURE 4:**
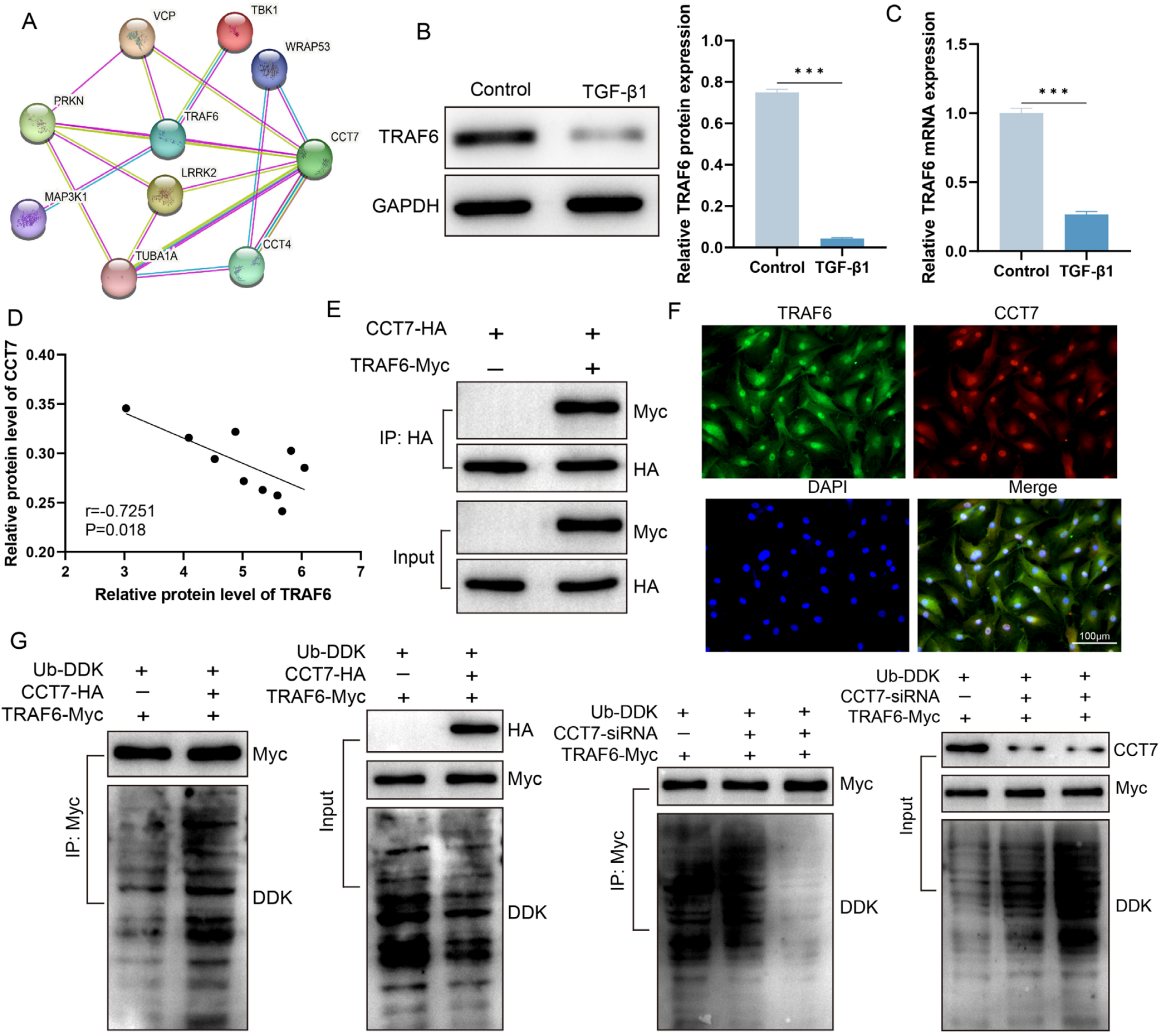
CCT7 regulates the stability of TRAF6 through ubiquitination. (A) Protein–protein interaction (PPI) analysis showing the interaction between CCT7 and TRAF6. (B) Western blot analysis of TRAF6 protein expression in fibroblasts treated with TGF‐β1. (C) qPCR analysis of TRAF6 mRNA expression in fibroblasts treated with TGF‐β1. (D) Correlation analysis of CCT7 and TRAF6 expression in fibroblasts. (E) Coimmunoprecipitation (Co‐IP) analysis confirming the interaction between CCT7 and TRAF6. (F) Immunofluorescence colocalisation of CCT7 and TRAF6 in fibroblasts. (G) Western blot analysis of TRAF6 ubiquitination in fibroblasts. ***p < 0.001.

We next investigated the molecular mechanism by which CCT7 regulates autophagy. Protein–protein interaction (PPI) analysis suggested a potential interaction between CCT7 and TRAF6 (Figure 4A). qPCR and Western blot analyses demonstrated that TGF‐β1 treatment significantly decreased the mRNA and protein expression of TRAF6 (Figure 4B,C). Moreover, correlation analysis demonstrated that CCT7 expression was negatively correlated with TRAF6 expression levels (Figure 4D). To verify the interaction between CCT7 and TRAF6, we performed Co‐IP experiments, which demonstrated their interaction (Figure 4E). Additionally, immunofluorescence colocalisation results indicated that TRAF6 and CCT7 colocalised in fibroblasts (Figure 4F). In order to determine whether CCT7 affects the ubiquitination of TRAF6, we conducted western blot analysis of ubiquitinated TRAF6. The results revealed that CCT7 regulates the stability of TRAF6 through ubiquitination (Figure 4G).

### 3.5 Knockdown of TRAF6 Can Reverse the Effect of CCT7 Knockdown on Fibroblast Autophagy

**FIGURE 5:**
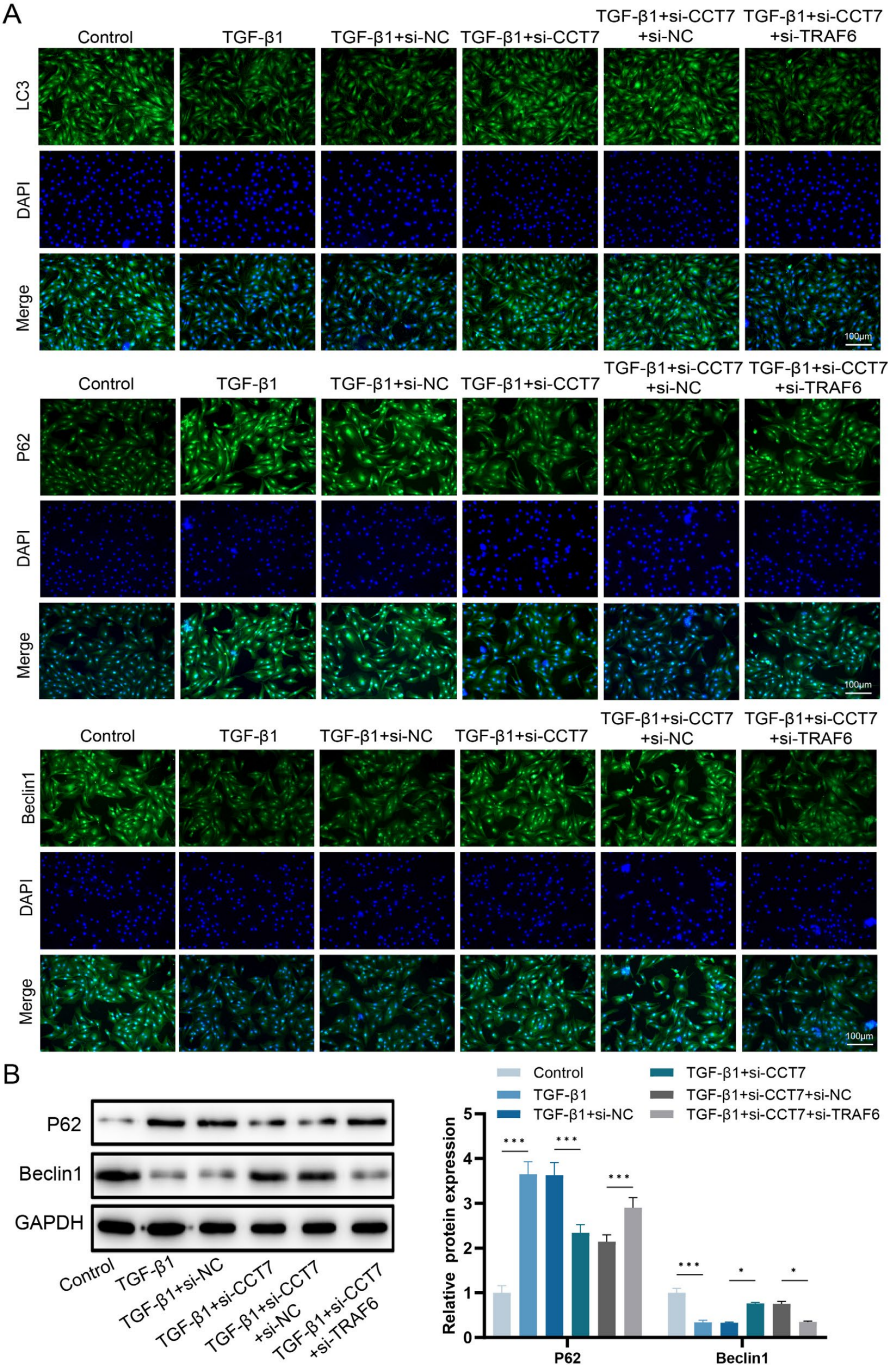
Knockdown of TRAF6 reverses the effects of CCT7 knockdown on fibroblast autophagy. (A) Immunofluorescence staining of LC3, p62 and Beclin‐1 in fibroblasts, which were cotransfected with *CCT7* siRNA and *TRAF6* siRNA. (B) Western blot analysis of p62 and Beclin‐1 protein expression in fibroblasts, which were cotransfected with *CCT7* siRNA and *TRAF6* siRNA. *p < 0.05, ***p < 0.001.

To further verify whether the effect of CCT7 on fibroblast autophagy is mediated by regulating the stability of TRAF6, we cotransfected fibroblasts with CCT7 siRNA and TRAF6 siRNA. Immunofluorescence and Western blot analyses showed that CCT7 knockdown significantly increased the levels of LC3‐II and Beclin‐1, while decreasing the expression of p62. Notably, these effects were reversed following TRAF6 silencing (Figure 5A,B).

### 3.6 Knockdown of TRAF6 Can Reverse the Effect of CCT7 Knockdown on the Expression of Fibrotic Markers in Fibroblasts

**FIGURE 6:**
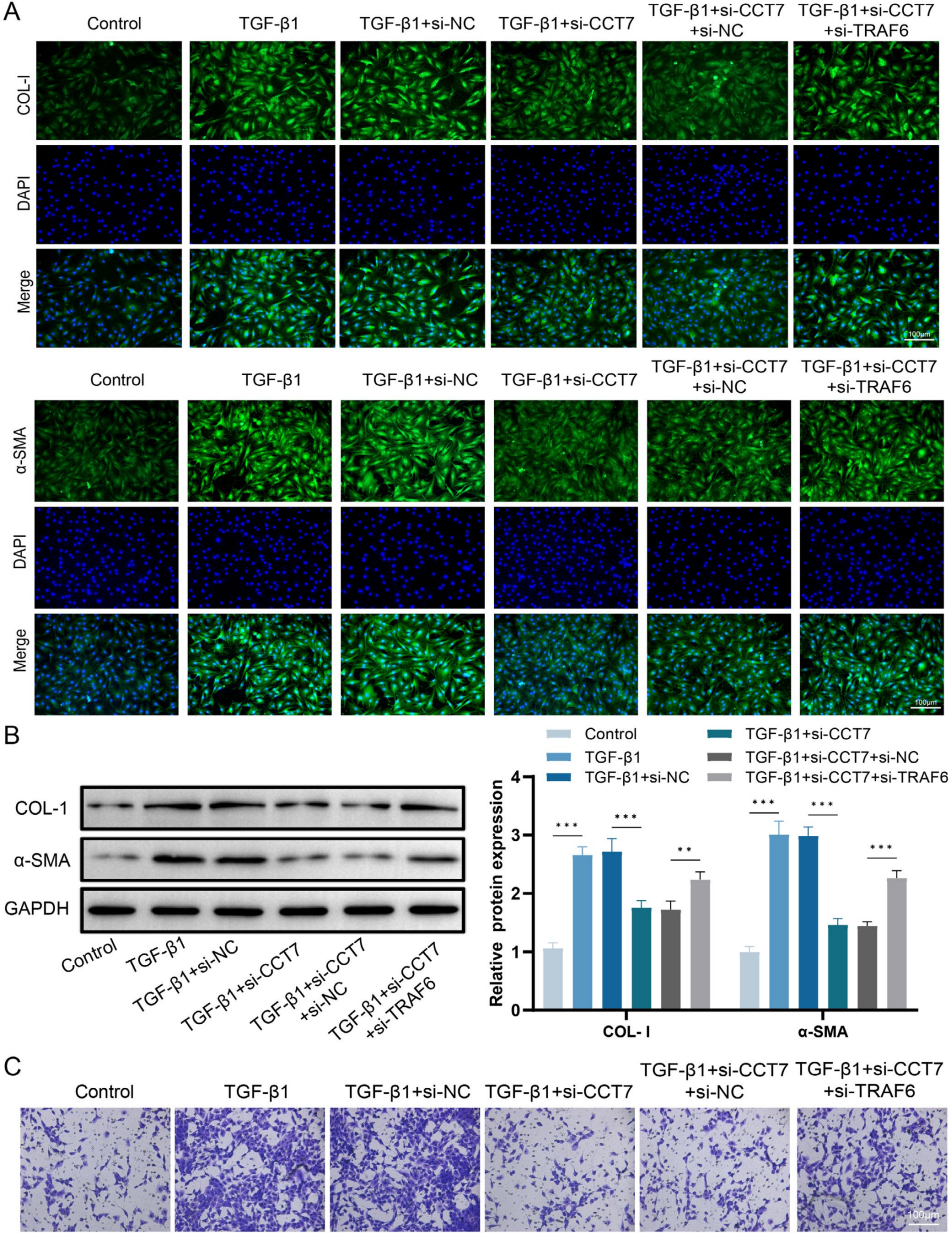
Knockdown of TRAF6 reverses the effect of CCT7 knockdown on the expression of fibrotic markers in fibroblasts. (A) Immunofluorescence staining of COL‐I and α‐SMA in fibroblasts, which were cotransfected with *CCT7* siRNA and *TRAF6* siRNA. (B) Western blot analysis of COL‐I and α‐SMA expression levels in fibroblasts, which were cotransfected with *CCT7* siRNA and *TRAF6* siRNA. (C) Transwell assay showing the migratory capacity of fibroblasts, which were cotransfected with *CCT7* siRNA and *TRAF6* siRNA. **p < 0.01, ***p < 0.001.

We further evaluated the effect of TRAF6 knockdown on the expression of fibrotic markers in cells. Our results demonstrated that TRAF6 knockdown significantly attenuated the inhibitory effect of CCT7 silencing on the expressions of α‐SMA and COL‐I (Figure 6A,B). Additionally, Transwell assays confirmed that TRAF6 knockdown remarkably enhanced the migratory capacity of CCT7‐silenced fibroblasts (Figure 6C). These data indicate that CCT7 exerts a pro‐fibrotic function by regulating TRAF6‐mediated autophagy.

### 3.7 Knockdown of CCT7 Alleviates Knee Joint Fibrosis in Rats by Promoting Autophagy

**FIGURE 7:**
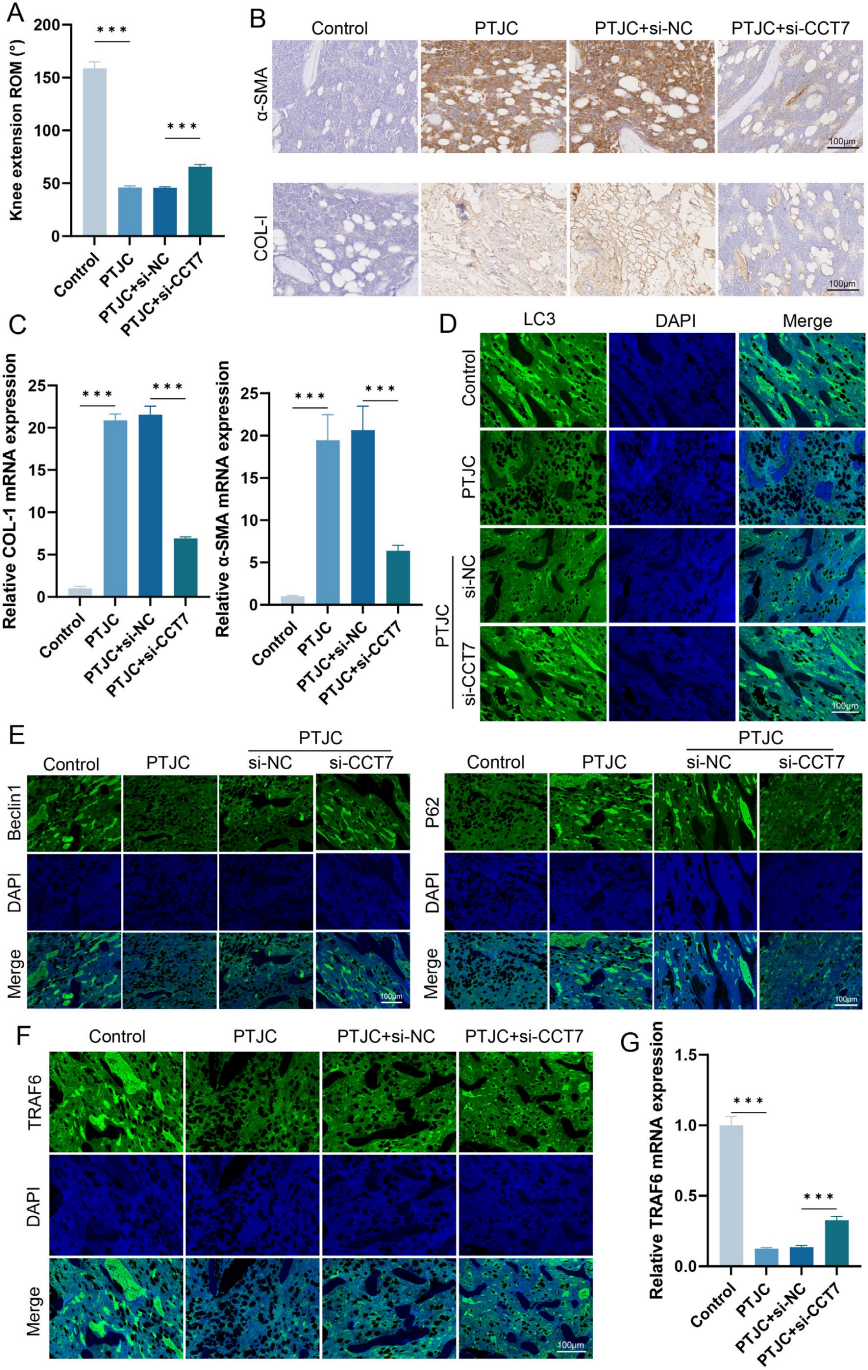
Knockdown of CCT7 promotes autophagy and reduces fibrosis in vivo. (A) Knee extension range of motion (ROM) in different treatment groups. (B) Representative immunohistochemical staining images of α‐SMA and COL‐I in joint tissue from different treatment groups. (C) qPCR analysis of α‐SMA and COL‐I expression in joint tissue from different treatment groups. (D‐E) Immunofluorescence staining of LC3, p62 and Beclin‐1 in joint tissue from different treatment groups. (F) Immunofluorescence staining of TRAF6 in joint tissue from different treatment groups. (G) qPCR analysis of TRAF6 expression in joint tissue from different treatment groups. ***p < 0.001.

Finally, we evaluated the effects of CCT7 knockdown on fibrosis and autophagy in animal models. Knee extension ROM was measured following arthrotomy, revealing a significant decrease in the PTJC group compared with controls, which was notably ameliorated by CCT7 knockdown (Figure 7A). Immunohistochemical analysis showed that compared with the control group, the expression of α‐SMA and COL‐I was significantly elevated in the model group. However, these increases were markedly attenuated in the PTJC group treated with si‐CCT7 (Figure 7B). The qPCR results were consistent with the immunohistochemical findings (Figure 7C). Subsequently, we detected the expression of autophagy‐related markers. Immunofluorescence analysis revealed that compared with the control group, the model group exhibited decreased expressions of LC3‐II and Beclin‐1, along with increased p62 expression. Notably, CCT7 knockdown reversed these changes (Figure 7D,E). Additionally, we evaluated the expression of TRAF6. qPCR and immunofluorescence analyses demonstrated that TRAF6 expression was significantly upregulated in the PTJC + si‐CCT7 group compared with the PTJC group (Figure 7F,G). These data indicate that CCT7 alleviates knee joint fibrosis following PTJC in rats by regulating TRAF6‐mediated autophagy.

## 4 Discussion

In this study, we found that CCT7 expression was significantly increased in the fibrotic joint capsule of rats following PTJC in a rat model. Further in vitro studies demonstrated that CCT7 knockdown inhibited the expression of fibrotic markers induced by TGF‐β1 in fibroblasts and promoted cellular autophagy. Mechanically, CCT7 regulates the degradation of TRAF6 through ubiquitination. Knockdown of TRAF6 can reverse the promoting effect of CCT7 knockdown on autophagy and promote the TGF‐β1‐induced fibrotic process. The results of animal experiments support this finding at the cellular level, indicating that CCT7 may serve as a potential therapeutic target for PTJC.

CCT7 is a subunit of the chaperonin complex involved in the folding and unfolding of cytoskeletal proteins, such as tubulins and actins. This complex is composed of two identical stacked rings, each containing eight distinct proteins. Unfolded polypeptides enter the central cavity of the complex and are folded in an ATP‐dependent manner [21, 22]. During fibrosis, cells face persistent stress, leading to the buildup of unfolded or misfolded proteins. As a result, cells typically activate the unfolded protein response (UPR) to maintain protein homeostasis [23]. CCT7, as a key subunit of the chaperone complex, may play an important role in this process. He et al. [11] found that CCT7 was overexpressed in fibroblasts from a rat model of PTJC, and that inhibition of CCT7 significantly exerted antifibrotic effects. Yi et al. [13] analysed the bioactivity of CCT subunits in fibroblasts from the same model, suggesting that these subunits may serve as potential molecular biomarkers and therapeutic targets for novel PTJC treatments. In our study, we observed that fibrotic markers such as TGF‐β1, α‐SMA and COL‐I were significantly upregulated in joint tissues, along with an increased expression of CCT7. This finding indicates that CCT7 may be closely associated with the development of PTJC. Additionally, research by Latha Satish et al. [14] demonstrated that the eta subunit of the chaperonin‐containing T‐complex polypeptide (CCT‐eta) is a specific regulator of fibroblast motility and contraction. He et al. [11] found that siRNA‐mediated downregulation of CCT‐eta inhibits CCT‐eta and α‐SMA expression, and reduces fibroblast functionality, including cell migration rate and collagen synthesis. As expected, our research results demonstrate that knockdown of CCT7 significantly suppresses the fibrotic phenotype of fibroblasts, including a reduction in the expression of α‐SMA and COL‐I, as well as decreased cell migratory capacity. This indicates that CCT7 plays a critical role in regulating fibroblast activation and the progression of fibrosis.

Further research revealed a PPI‐predicted interaction between CCT7 and TRAF6. Our experimental data confirmed this, with Co‐IP and immunofluorescence colocalisation assays both indicating an interaction between CCT7 and TRAF6. As a key signalling protein, TRAF6 is involved in multiple cellular processes, including inflammatory responses, cell survival and autophagy. Recent studies have shown that TRIB3 can inhibit the development of pulmonary fibrosis by regulating TRAF6 ubiquitination or increasing TRAF6 expression [24]. Xu et al. found that ABL1 modulates inflammation in pneumonia by inhibiting the phosphorylation of IκBα and p38 and regulating TRAF6 ubiquitination [25]. Zhang et al. discovered that Rnd3 inhibits endothelial pyroptosis in atherosclerosis by regulating TRAF6 ubiquitination [26]. In this study, we found that CCT7 can modulate TRAF6 ubiquitination. Their interaction in fibroblasts leads to reduced TRAF6 expression, and we observed that the binding of CCT7 to TRAF6 accelerates TRAF6 ubiquitination and degradation. This suggests that CCT7 may influence TRAF6's ubiquitination state, altering its intracellular localisation and function, and thus affecting the activation of downstream signalling pathways.

Autophagy is a highly conserved catabolic process in eukaryotic cells. It degrades cytoplasmic components, abnormal proteins and dysfunctional organelles via lysosomes, thereby maintaining cellular homeostasis and cell survival [27, 28]. However, its dysregulation has been implicated in fibrotic diseases across tissues [29]. For example, in hepatic fibrosis, impaired autophagy promotes hepatic stellate cell activation and collagen deposition [30, 31], while in pulmonary fibrosis, autophagy deficiency exacerbates alveolar epithelial cell apoptosis and ECM accumulation [32]. Our findings are consistent with these studies. In the joint capsule tissue after PTJC, we observed an increased expression of p62 and decreased expression of LC3‐II and Beclin‐1, suggesting the presence of an autophagy defect. Notably, CCT7 knockdown restored autophagic flux and reduced fibrosis, as indicated by elevated LC3‐II and Beclin‐1 expression, decreased p62 expression and decreased expression of fibrosis markers. These results indicate that autophagy reactivation may counteract the transition of fibroblasts to myofibroblasts, a hallmark of fibrosis. TRAF6, an E3 ubiquitin ligase, is central to autophagy regulation. Yin et al. [33] found that TRAF6 promotes autophagy by ubiquitinating Beclin‐1, while PRDX1 negatively regulates TLR4 signalling‐induced NFKB activation and autophagy by inhibiting TRAF6 ubiquitin ligase activity. Kin et al. [34] demonstrated that TRAF6 interacts with Beclin‐1 and induces its ubiquitination, thereby promoting autophagy. In this study, autophagy activity was found to be inhibited in the posterior joint capsules of PTJC rats, which may be associated with reduced Beclin‐1 ubiquitination mediated by TRAF6. In the PTJC rat model, elevated levels of CCT7 decrease TRAF6 protein stability by promoting its ubiquitination, thereby disrupting TRAF6‐mediated K63‐linked ubiquitination of Beclin‐1, ultimately resulting in diminished autophagy activity. These findings suggest that modulating TRAF6 expression or activity to enhance fibroblast autophagic capacity may serve as an effective strategy for mitigating joint fibrosis.

## 5 Conclusion

In conclusion, our research findings suggest that CCT7 may serve as a key regulatory factor in joint fibrosis. Specifically, CCT7 plays a critical role in modulating TRAF6‐mediated autophagy, a regulatory mechanism that helps alleviate PTJC. These insights highlight CCT7 as a promising therapeutic target for the treatment of PTJS.

## Author Contributions

Yutai Lia wrote the manuscript. Wenhui Zhang and Wenbin Xu performed the experiments. Yuliang Huanga and Honghua Caia analyzed and interpreted the data. Nanwei Zhanga and Tangzhao Liang contributed reagents, materials, and analysis tools. Tangzhao Liang and Guihua Liu designed the study and revised the manuscript.

## Funding

This research was supported by Guangdong Natural Science Foundation, Basic and Applied Basic Research Fund, Huizhou Joint Fund (Guangdong‐Huizhou Regional Joint Fund—Regional Cultivation Project) (2022A1515140189, 2023A1515140185); Guangdong Natural Science Foundation—General Program (2023A1515010386); National Natural Science Foundation Project (82372402).

## Ethics Statement

Animal experiments were approved by the Ethics Committee of Guangzhou Seyotin Biotechnology Co. Ltd (SYT2024112).

## Conflicts of Interest

The authors declare no conflicts of interest.

## Data Availability

The data that support the findings of this study are available from the corresponding author upon reasonable request.
